# Deep-sea Sediment Resuspension by Internal Solitary Waves in the Northern South China Sea

**DOI:** 10.1038/s41598-019-47886-y

**Published:** 2019-08-20

**Authors:** Yonggang Jia, Zhuangcai Tian, Xuefa Shi, J. Paul Liu, Jiangxin Chen, Xiaolei Liu, Ruijie Ye, Ziyin Ren, Jiwei Tian

**Affiliations:** 1 Shandong Provincial Key Laboratory of Marine Environment and Geological Engineering, Ocean University of China, Qingdao, 266100 P.R. China; 2 Laboratory for Marine Geology, Qingdao National Laboratory for Marine Science and Technology, Qingdao, 266061 P.R. China; 3 Key Laboratory of Marine Sedimentology and Environmental Geology, First Institute of Oceanography, State Oceanic Administration, Qingdao, Shandong 266061 China; 4 Department of Marine, Earth and Atmospheric Sciences, North Carolina State University, Raleigh, NC 27695 USA; 5 The Key Laboratory of Gas Hydrate, Ministry of Natural Resources, Qingdao Institute of Marine Geology, Qingdao, 266071 P.R. China; 6 Physical Oceanography Laboratory/CIMST, Ocean University of China, Qingdao, 266100 P.R. China; 7 Key Laboratory of Marine Environment & Ecology, Ministry of Education, Qingdao, 266100 P.R. China

**Keywords:** Physical oceanography, Ocean sciences

## Abstract

Internal solitary waves (ISWs) can cause strong vertical and horizontal currents and turbulent mixing in the ocean. These processes affect sediment and pollutant transport, acoustic transmissions and man-made structures in the shallow and deep oceans. Previous studies of the role of ISWs in suspending seafloor sediments and forming marine nepheloid layers were mainly conducted in shallow-water environments. In summer 2017, we observed at least four thick (70–140 m) benthic nepheloid layers (BNLs) at water depths between 956 and 1545 m over continental slopes in the northern South China Sea. We found there was a good correlation between the timing of the ISW packet and variations of the deepwater suspended sediment concentration (SSC). At a depth of 956 m, when the ISW arrived, the near-bottom SSC rapidly increased by two orders of magnitude to 0.62 mg/l at 8 m above the bottom. At two much deeper stations, the ISW-induced horizontal velocity reached 59.6–79.3 cm/s, which was one order of magnitude more than the seafloor contour currents velocity. The SSC, 10 m above the sea floor, rapidly increased to 0.10 mg/l (depth of 1545 m) and 1.25 mg/l (depth of 1252 m). In this study, we found that ISWs could suspend much more sediments on deepwater areas than previously thought. Specifically, we estimated that ISWs could induce and suspend 787 Mt/yr of sediment from shelf to deep-sea areas of the northern South China Sea. The total amount of sediment resuspended by shoaling ISWs was 2.7 times that of river-derived sediment reaching the northern South China Sea. This accounted for 6.1% of the global river-discharged sediment (16.4% of that from Asian rivers) transported to the sea.

## Introduction

Internal solitary waves (ISWs), or internal solitons, are formed in stratified oceans and create remarkable interactions between ocean currents and the seafloor in a relatively short time (tens of minutes)^1,2^. The interactions between ISWs and the seabed are considered important factors that promote sediment resuspension and transport, as well as seabed deformation and instability^3–5^. Notably, these interactions have led to sand waves and scour channels along the shelf break of the northern South China Sea^5,6^.

ISWs can cause sediment resuspension and transport in the shoaling process^7–10^, and large-scale suspension can erode seafloor sediments and form marine nepheloid layers^6,11–13^. Moreover, ISWs can even shape the slope morphology and affect the sedimentary landscape of the seabed^3,4,14–16^. Sediment resuspension events triggered by ISWs have been observed and studied in marginal seas, shelf and subaqueous delta areas, such as the Middle Atlantic Bight^17^, the western Portuguese mid-shelf^18^, Otsuchi Bay^12^, the northern South China Sea^5,19^, the Oregon shelf ^20^, Massachusetts Bay^21^, the California shelf ^22^, and New York Bay^23^. The benthic nepheloid layers (BNLs) and intermediate nepheloid layer (INLs) formed by shoaling ISWs have been widely observed in the field^6,11,13,18^. Previous studies of ISW-induced sediments resuspension have mainly concentrated on shallow sea areas with water depths of less than 200 m. The only deep-sea study ever reported was conducted in the continental shelf break area of the northern South China Sea^6^. Reeder *et al*. (2011) observed that ISWs disturbed the seabed sediments and formed 200-m-thick BNLs. However, the results lacked near-bottom flow evidence and have been interpreted in multiple ways^5^. Additionally, the amount of suspended sediment has never been directly measured nor convincingly estimated.

ISWs impact the ocean at all depths, but deeper areas have not been studied in detail. The ISWs in the South China Sea are the largest amplitude waves in the world; these waves occur in the tidal period, up to twice a day, and generally in the form of wave packets^24^. Here, ubiquitous ISWs are believed to generate in the Luzon Strait and propagate in the WNW direction, eventually dissipating in the shallow coastal sea, spreading more than 600 kilometres^25^. Existing studies have concentrated on the Dongsha sea^6,19,24^, and few field attempts have been made to describe seabed sediment resuspension on other foot prints of ISWs. However, with seismic oceanography methods, recent studies have shown that ISWs can reach and shape the seafloor to depths of 400 m or more^26,27^, causing potentially substantial sediment resuspension near the seafloor. To address these questions, we studied ISW-induced sediment resuspension based on *in situ* observations and investigations of marine nepheloid layers. We observed at least four thick (70–140 m) BNLs at water depths of approximately 956–1545 m. Furthermore, we found a good correlation between the timing of the ISW packet and the variations in the deepwater suspended sediment concentration (SSC).

## Methods

### Measurements from the mooring system

**Figure 1:**
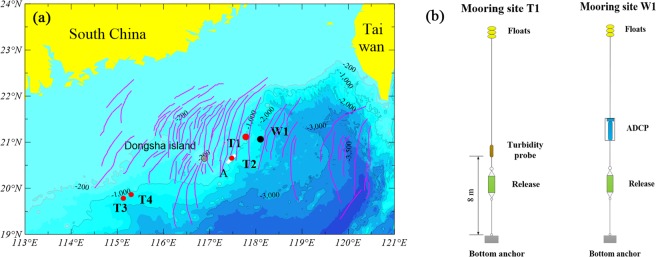
Location and design of the mooring system and bathymetric map of the northern South China Sea. (**a**) The pink curves represent the ISWs observed in satellite and ASAR images^44^, which are the largest amplitude waves in the world^2,45^. Common conditions for the propagation of ISWs on the surface are illustrated, and most ISWs propagate in the WNW direction. The white outlines denote the regions where bottom nepheloid layers were observed by the multibeam swath bathymetric system. W1 and T1 are the locations of the mooring system. T2, T3 and T4 are the locations that were used for In situ investigation. Bathymetry data are generated by Surfer 12.0 (Golden Software, Inc.) and are downloaded from https://www.ngdc.noaa.gov/mgg/global/. (**b**) The vertical mooring structure at sites W1 and T1.

A deepwater mooring system (T1) was deployed at a depth of 956 m (21°05′N, 117°50′E) in the northern South China Sea for nearly 8 consecutive days from June 19th to June 27th, 2017 (Fig. 1). The mooring system was equipped with a turbidity probe on the compact RBR concerto Tu (concerto, RBR, Canada) 8 m above bottom (mab). Turbidity data were collected every 10 seconds. Another deepwater mooring system (W1) was deployed at approximately 2,000 m (21°01′N, 118°09′E) depth in the northern South China Sea for nearly 1 consecutive year from June 2016 to June 2017 (Fig. 1), with retrieval and redeployment in June 2017. This mooring system was equipped with an upward-looking acoustic doppler current profiler (ADCP-LR75) (Workhorse 75 K, TRDI, America). The ADCP-LR75 sampled velocities every 2 min at an 8-m-bin vertical resolution to obtain measurements from approximately 0 m to 1000 m.

### Marine nepheloid layer identification

Backscattering data were acquired aboard *Dongfanghong2* ship using a hull-mounted multibeam swath bathymetric system (12-kHz, Kongsberg Simrad EM302). Tracklines provided 120–150% seafloor coverage. Using QPS Fledermaus Midwater software (QPS B.V. Netherlands), the water column backscattering data were evaluated parallel and perpendicular to the tracklines to identify water column anomalies.

### Water column profile

The profile of water column temperature, salinity, the turbidity and velocity was measured by a conductivity–temperature–depth (CTD) profiling system (SBE 9/11 plus, Seabird, America) assembling with a lowered-ADCP (300 K, TRDI, America) at T2 (depth of 967 m; 117°35′E, 20°44′N), T3 (depth 1545 m; 19°48′N, 115°05′E) and T4 (depth 1252 m; 19°57′N, 115°18′E). The probe extended to 10 m above the seafloor and kept near the seafloor for some time.

### Estimate of SSC from turbidity

Selective sediment sampling was implemented nearly the mooring sites to determine the relationship between turbidity (NTU) and the actual SSC (mg/l). Sediments were collected on 142-mm membrane filters (4 µm Nuclepore). Using the least square fitting method, a proportional linear relation between the turbidity recorded by an RBR and the SSC was determined, where SSC (mg/l) = 0.77 × turbidity (NTU). The standard deviation of the error in predicting the SSC was 0.01.

## Results

### Marine nepheloid layers formed by ISWs

**Figure 2:**
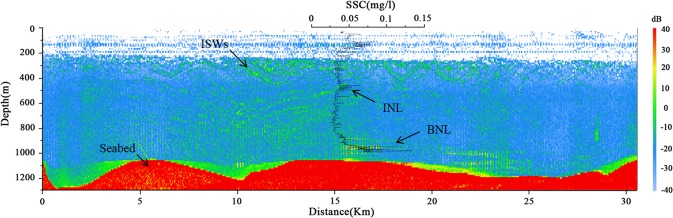
The water column structure of the ISW with a surface expression at “A” in Fig. 1. The data were collected over a period of approximately 2 h using a hull-mounted multibeam swath bathymetric system as the ship moved along the transect to intercept the incoming trans-basin ISW. The ISW packet propagated along the thermocline approximately 200 m in the form of a large depressional wave. The suspended sediment concentration (SSC) profile was measured by a SBE9/11 plus CTD with an RBR concerto Tu sensor 10 m above the seafloor.

We used multibeam data acquired by the *Dongfanghong2* ship to investigate the marine nepheloid layer in summer 2017. The route was from Dongsha to Shenhu along the mid-continental slope. The BNLs were produced by the resuspension of seabed sediments^4,28,29^ and BNLs formed by shoaling ISWs have been observed in the continental shelf break of Dongsha^6^. These particles in the BNL have a lifetime of days, and allow for appreciable lateral advection^28,29^. During our observation period, one BNL formed by ISWs was identified and named “A” (Figs 1a and 2). The ISW packet propagated along the thermocline approximately 200 m in the form of a large depressional wave. At the same time, we measured the SSC profile by CTD with an RBR concerto Tu sensor at T2 (depth of 967 m; 117°35′E, 20°44′N) of “A”. The thickness of the BNL was consistent with the measured SSC, with a maximum thickness and concentration of 70 m and 0.14 mg/l, respectively (Fig. 2). McCave (1986) indicated that the highest SSC occur in BNLs and typically range from 0.1–0.5 mg/l^29^. What we observed on the bottom was thought to be a BNL. In addition, one weak INL appeared above the BNL, with a thickness of 50 m. The multibeam data corresponded to the concentration profile of the water column and displayed high concentrations. There were ubiquitous BNL above the seabed along the transect, especially in the scour channels (Fig. 2). These data showed that ISWs affected deeper water environments (depth of 967 m), which was more than we previously thought.

### *In situ* observations of ISW-induced sediment resuspension

**Figure 3:**
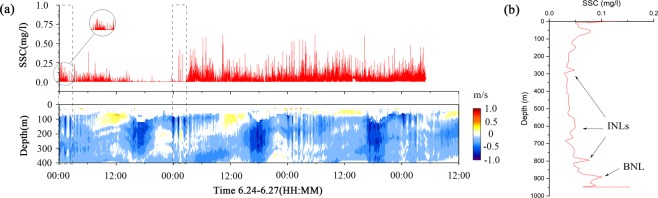
(**a**) Time series of the suspended sediment concentration from 00:00 on June 24th to 12:00 on June 27th (Beijing time) at 8 m above bottom (mab) and the velocity (EW) measured by the upward-looking ADCP at the moorings. The two moorings were deployed along the ISW propagation path (see Fig. 1a). The time interval was approximately 7 hours between the two moorings according to the velocity of the ISW at W1. The effect of the time difference between the two moorings was negated by normalization. Data of SSC were not available for approximately 7 hours on June 27th because mooring T1 was reclaimed before T2. Black dashed square indicated two periods in which the SSC was remarkably consistent with the ISW events. (**b**) The suspended sediment concentration (SSC) profile in the reclamation process on June 27th as the ISW packet passed.

*In situ* observations focused on the continental slopes of Dongsha, where seabed sediment resuspension has been observed in the continental shelf break^6^. We deployed two moorings along the ISW propagation path to observe changes in the velocity field at W1 (2000 m; 21°01′N, 118° 09′E) and ISW-induced sediment resuspension and the BNL thickness at T1 (956 m; 21° 05′N, 117° 50′E) (Fig. 1). We found that the near-bottom SSC, 8 m above bottom (mab), oscillated around a background concentration of 0.004 mg/l (Fig. 3a). During the SSC measurements, the apparent ISW packet appeared on June 24th. The correlation between the SSC recorded by the RBR concerto Tu (concerto, RBR, Canada) at T1 and the velocities recorded by the ADCP (LR75, TRDI, America) at W1 was shown in Fig. 3 from 00:00 on June 24th to 12:00 on June 27th (Beijing time). We identified four events associated with the ISW packet and three internal tides (Fig. 3a). The data indicated that the increases in both the baseline and peak SSC values were associated with ISW events. There were two periods in which the SSC was remarkably consistent with the ISW events (Fig. 3a), and the maximum SSC was 0.62 mg/l. These findings indicated that the increase in the SSC was caused by the ISW interacting with the seabed sediments in the depth of 956 m. The SSC increased by two orders of magnitude to a maximum value, and the degree of suspension was intense.

The SSC did not immediately decline after the ISW packet passed and continued for several hours to form the BNL. After 03:00 on June 25th, the BNL remained intact (Fig. 3a). Shoaling ISWs could erode, resuspend and transport suspended sediment offshore to marine nepheloid layers along continental shelves and into deep-sea basins^11,12,30^. Therefore, the suspended sediment on the slope moved downward, resulting in a continuously high SSC (Fig. 3a). This was the reason that the SSC peaked after the ISW packet passed. Although downward movement of the resuspended sediment may have been related to internal tides, it still had a minor influence on the BNL from June 25th to 26th than ISW. When the T1 mooring was reclaimed on June 27th, the SSC profile was measured as the ISW packet passed. The SSC profile indicated that one BNL formed with a thickness of 100 m (Fig. 3b), and the maximum concentration was 0.15 mg/l. Moreover, three weak INLs formed above the BNL at approximately 300 m, 600 m and 800 m. The INLs may have been formed by the suspended sediment moving downward.

### *In situ* investigation of ISW-induced sediment resuspension

**Figure 4:**
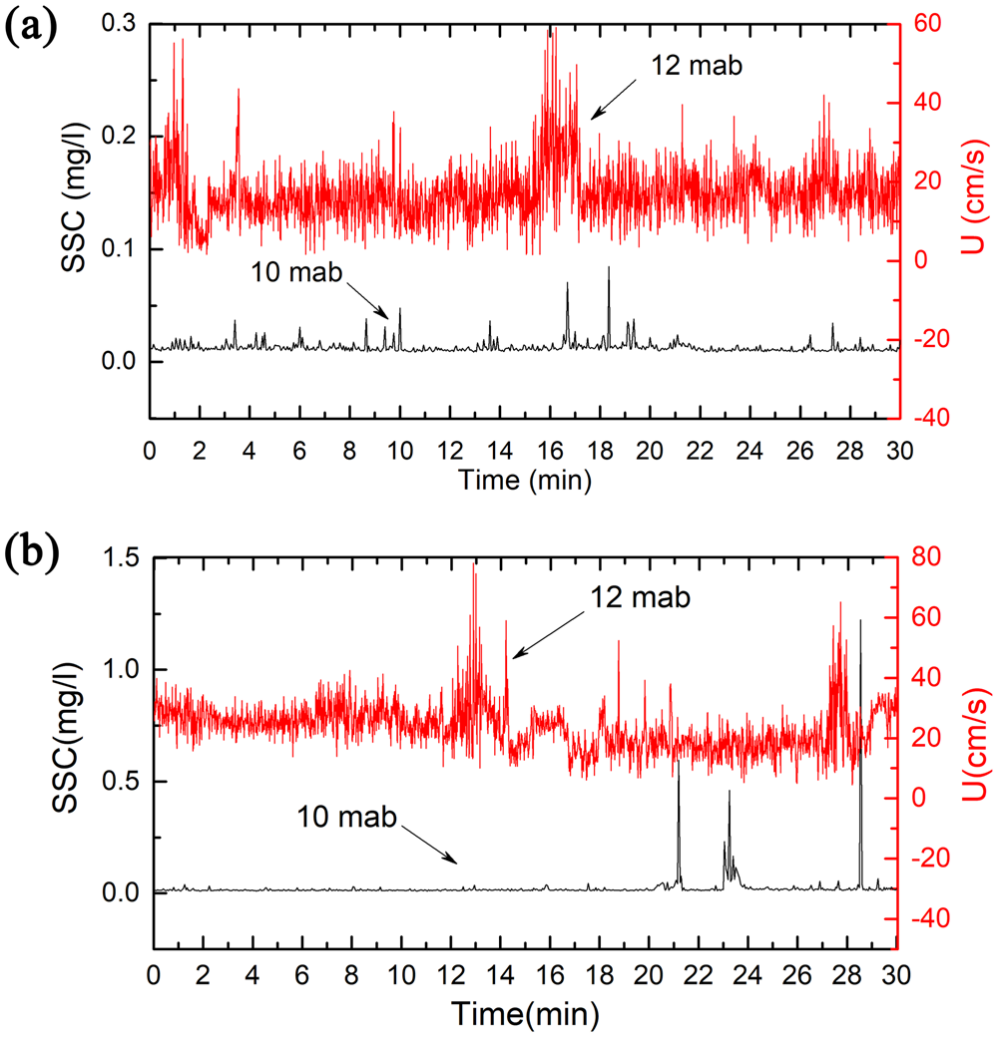
Time series of the suspended sediment concentration (SSC) 10 m above bottom (mab), and the mean horizontal velocity (U) measured by lowered-ADCP on the SBE9/11 plus CTD 12 m above bottom (mab). Due to missing bottom tracking, we replaced it with GPS of ship. (**a**) T3 and (**b**) T4 were corresponding to red dashed square in Fig. 5(a,b), respectively.

**Figure 5:**
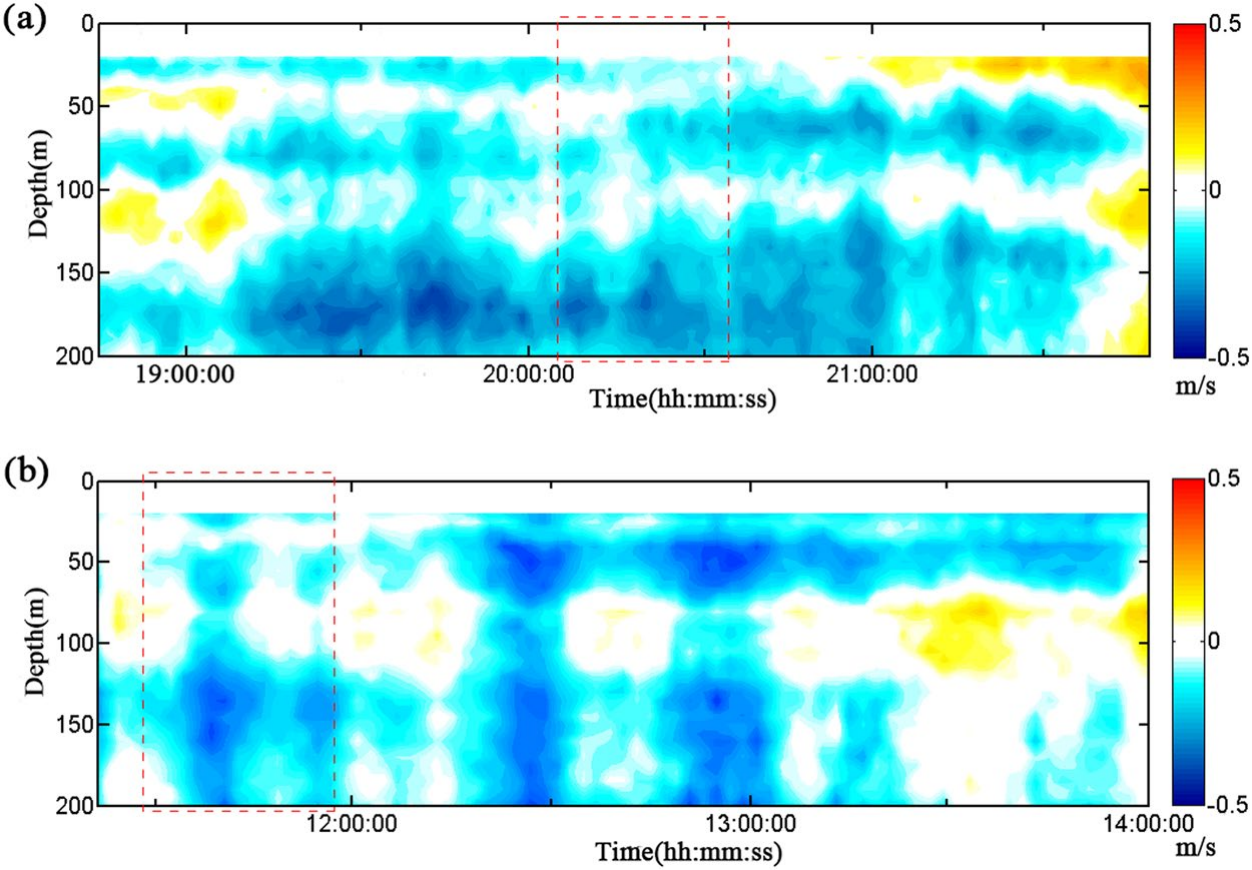
Velocity (E-W) for depth zones above 200 m measured using a hull-mounted ADCP: (**a**) T3 is located at 19°48′N,115°05′E at a depth of 1545 m and (**b**) T4 is located at 19°57′N, 115°18′E at a depth of 1252 m (Fig. 1). Red dashed square indicated the period included suspended sediment concentration and the mean velocity near the seabed in Fig. 4.

We made in situ investigation of the continental slopes in Shenhu, where few seabed sediment resuspension events have been described^5^. We measured the SSC and velocity near the seafloor at T3 (depth 1545 m; 19°48′N, 115°05′E) and at T4 (depth 1252 m; 19°57′N, 115°18′E) using the CTD assembling with a lowered-ADCP (Fig. 1). During the observation period, the near-seabed SSC at 10 mab oscillated around 0.01 mg/l (Fig. 4). The near-seabed mean horizontal velocity measured by the lowered-ADCP at 12 mab at T3 and T4 oscillated around the baseline value of ~20 cm/s (Fig. 4), which was one order of magnitude larger than the velocity of contour current at the seafloor^31^. Additionally, the mean horizontal velocities at T3 and T4 displayed several peak values, and the maximums reached 59.6 cm/s and 79.3 cm/s, respectively, which reflected episodic events associated with the ISWs (Fig. 4). The ISWs observed by the ADCP on the ship corresponded to the peak velocity values near the seafloor measured by the lowered-ADCP (Figs 4 and 5). When the ISW arrived, the near-seabed SSC rapidly increased by one order of magnitude to 0.10 mg/l at T3 and two orders of magnitude to 1.25 mg/l at T4 (Fig. 4). Moreover, some peak velocity values did not correspond to resuspension (Fig. 4b). The possible explanations were that sediment resuspension was a random phenomenon that was not only influenced by velocity. However, the sediment resuspension generally corresponded to ISWs.

**Figure 6:**
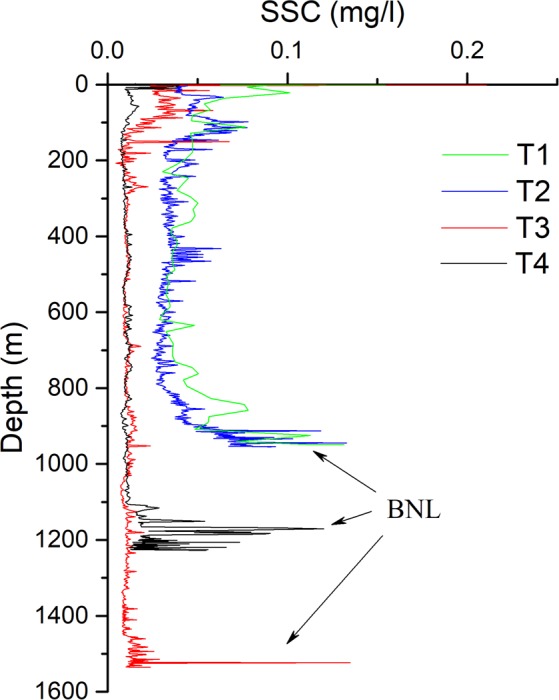
The suspended sediment concentration (SSC) profiles of water column at T1, T2, T3 and T4 (see Fig. 1 for their detail location). Every station has a thick BNL near bottom in the continental slope.

We observed the SSC profiles at T3 and T4 in the *in situ* investigation (Fig. 6). The SSC profiles at T3 and T4 displayed one thick BNL, and the thicknesses were approximately 90 m and 140 m, respectively. The maximum BNL concentrations were approximately 0.13 mg/l and 0.12 mg/l. The background concentrations in the water column were consistent at approximately 0.01 mg/l.

## Discussion

We found that the impact of the ISW in the deep-sea area was still large and formed thick BNLs in the maximum depth ~1500 m. The thickness of the BNLs formed by the ISW ranged from approximately 70–140 m (Figs 2, 3 and 6) along the continental slope. Considering the BNLs formed by shoaling ISWs at the continental shelf break^6^ and sediment resuspension along the continental shelf of the northern South China Sea^5,19,24^, the results suggest that ISWs can suspend sediments in their footprint areas as they propagate from continental slope to shelf regions.

The SSC profile indicated that there were weak INLs above the BNL at T1 and T2 in Dongsha (Figs 2, 3 and 6). However, there were no INLs found in Shenhu. Some studies have found that the marine nepheloid layers formed by shoaling ISWs offshore spread along continental shelves to the deep-sea basin^11,12,30^. A shallow-water BNL becomes an INL in the deep sea due to spreading from the continental shelf to the deep sea. The INLs in Dongsha might have been induced by the suspended sediment moving downward. The background concentration in the water column was four times higher in Dongsha (approximately 0.04 mg/l) than in Shenhu (approximately 0.01 mg/l) (Fig. 6). Based on observational data, Xu *et al*. (2012) found that when ISWs passed Dongsha, the mixing rate considerably increased^32^. Additionally, mixing was instantaneously enhanced, and several peaks were observed at different depths when the ISWs was about to arrive or had just passed^32^. The SSC profile indicated that mixing in the water column caused by ISWs was more significant in Dongsha than in Shenhu.

The mean horizontal velocity (at 12 mab) in the E-W and N-S directions measured by the lowered-ADCP oscillated around the baseline value of ~20 cm/s, and peak values reached 79.3 cm/s (Fig. 4). ISWs amplified bottom flow velocity passing rough topography, such as scour channel, and promoted the sediment resuspension^33–35^. Notably, the near-bottom velocity increased as the trough of the ISW approached in our field observation. Based on observations and analyses of sediment suspension events, it is generally believed that the near-bottom velocity is greater than 0.5 m/s, which could resuspend sediments^6^. Additionally, the velocity observed in this study was enough to suspend the seabed sediment. A near-bottom velocity of a similar order of magnitude was observed in the South China Sea^9,36^. Lien *et al*. (2014) even found that the near-bottom velocity exceeded 1.0 m/s as the trough of the ISW passed a continental shelf break^9^. The shoaling ISW induced a drastic near-bed current on the continental slope, which resulted in significant resuspension on the seafloor.

The average thickness and maximum concentration of the BNL formed by the ISW were approximately 100 m and 0.135 mg/l (Fig. 6), respectively. The footprint of the ISW that propagated from the continental slope to the shelf break of the northern South China Sea was approximately 1.62 × 10^11^ m^2^ (Supplementary Fig. S1). The statistical data suggest that there are more than 360 ISWs (around one ISW per day) each year in the northern South China Sea^37,38^. Therefore, we roughly estimated that the total amount of sediment resuspended by ISWs was approximately 7.87 × 10^8^ tons per year. The average thickness of BNL that we used to estimate this value was half of that reported by Reeder *et al*.^6^. We did not take the INL into account.

**Figure 7:**
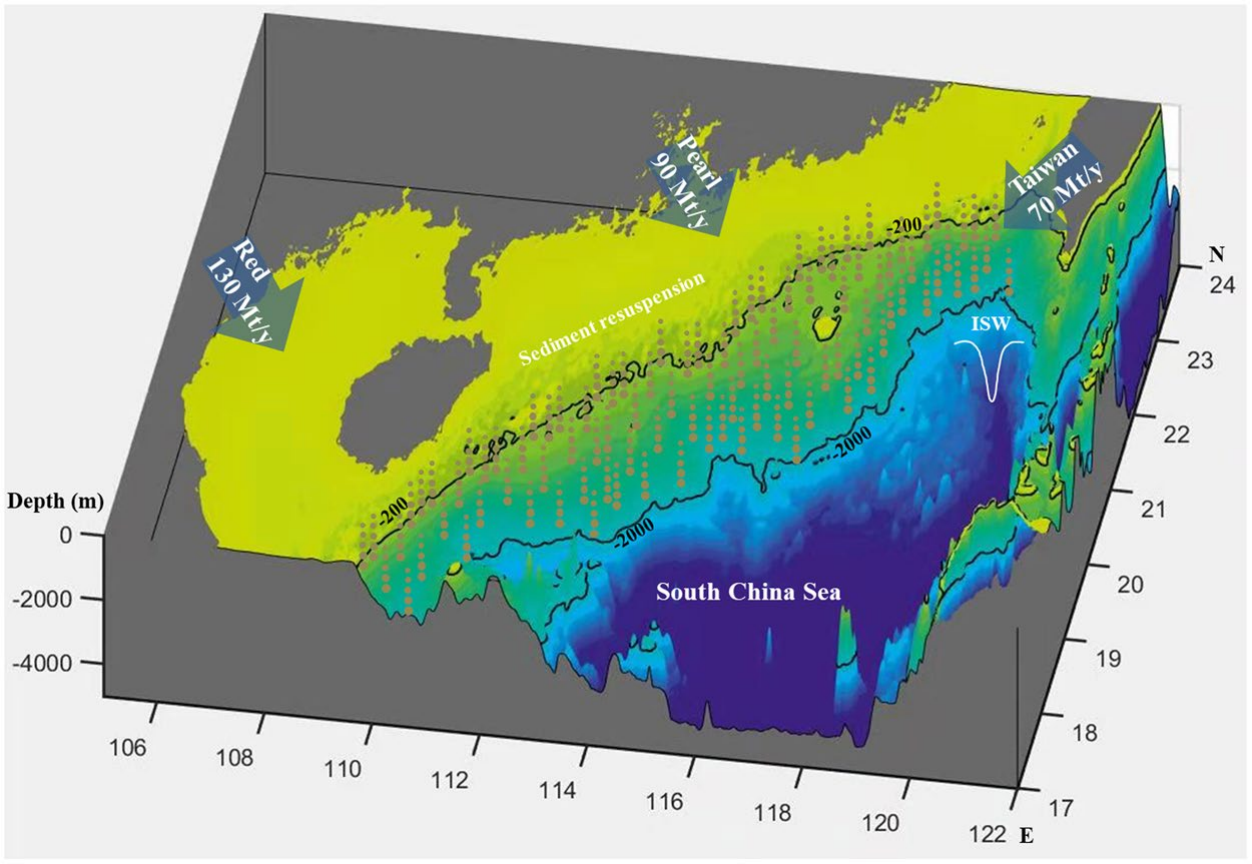
The three-dimensional topography map of the northeastern South China Sea and distribution of the mainly rivers and their historical annual sediment loads to the northern South China Sea mainly (Mt = million tons) (data from Liu *et al*., 2008; 2009). Sediments suspended by ISW in the footprints of ISW propagated from continental slope to shelf.

Recent studies have shown that the global flux of modern river-derived sediment reaching the coasts and oceans under Anthropocene conditions is approximately 12.8 × 10^9^ tons per year^39,40^, with a value of 4.8 × 10^9^ tons per year from Asian rivers^39^. The river-derived sediments that flow into the northern South China Sea are mainly from the Red River, Pearl River and some rivers in Taiwan^41^ (Fig. 7). The flux of river-derived sediment reaching the northern South China Sea is approximately 2.9 × 10^8^ tons per year^41,42^. The total amount of sediment resuspended by shoaling ISWs accounts for 6.1% of the global river-discharged sediment (16.4% of that from Asian rivers) transported to the sea and nearly three times the flux of river-derived sediment reaching the northern South China Sea.

Previous work has discovered that ISWs may erode, resuspend and transport sediments by BNL and subsequently offshore through the generation of INLs^12,30^. The BNLs have a lifetime of days, allowing for appreciable lateral advection^28,43^. Lateral transport of BNLs can reach especially far and may enter the deep ocean from shallow areas^43^. INL was constituted by fine particles, and these fine particles in the INL had very low settling velocity (e.g. less than ~5*10^−6^ m/s), which helped INL exist longer lifetime^28,29^. BNLs and INLs are thought to be transport channels both for land-sourced material to the seafloor and for suspended particles from the ocean margin to the ocean interior^28,35^. Due to sediment suspended by shoaling ISWs from continental slope to shelf, we inferred that the major suspended sediment ultimately was transported to the basin of the South China Sea.

The amount of resuspended sediment in the seabed cannot be ignored in the global oceans. This analysis indicates that ISWs play a controlling role in deep-sea, sedimentary, dynamic processes and seafloor evolution. ISWs can erode the sediment surface and shape continental slopes and shelves, and finally transport suspended particles from the ocean margin to the ocean interior by forming BNLs or INLs.

## Conclusions

This study found that sediment resuspension by ISWs formed a BNL and enhanced velocity along a continental slope based on mooring system observations and measurements with an lowered-ADCP equipped on CTD. The results provided fundamental evidence that ISWs could suspend sediments in their footprints as they propagated from the continental slope to the shelf of the northern South China Sea. We estimated that ISWs could suspend 787 Mt/yr sediment from the shelf to the deep sea in the northern South China Sea, which accounted for 6.1% (16.4% of the contribution of Asian rivers) of the global river-discharged sediment transported to the sea. The total amount of sediment resuspended by shoaling ISWs was nearly three times that of river-derived sediment reaching the northern South China Sea.

There are very few studies that put emphasis on the role of ISWs in deep-sea sediment resuspension, sedimentary and dynamic processes. The results provide an extremely useful case study for similar sediment resuspension events. BNLs and INLs produced by the suspended sediments are ubiquitous in oceans worldwide. Our findings represent a quantitative study on the role of ISWs for sediment transport in the marginal sea that may lead to a better understanding of sedimentary processes and seafloor evolution over geologic time, which is critical for geohazard assessment. Although the South China Sea is morphologically peculiar, we believe that ISW-induced sediment resuspension and transport would occur worldwide and that they have not yet interpreted in this way. This mechanism could be extended to similar areas developed in other marginal seas, especially where ISW activities are frequent and sediment resuspension is violent. These findings reinforce the theory that the relationship between ISWs and sediment resuspension may have been underestimated.

## Supplementary information

supplementary

## References

[1] Apel JR, Stepanyants LA, Lynch JF (2007). Internal solitons in the ocean and theireffect on underwater sound. The Journal of the Acoustical Society of America.

[2] Huang, X. *et al*. An extreme internal solitary wave event observed in the northern South China Sea. *Scientific Reports* **6** (2016). 10.1038/srep30041 PMC4956752 27444063

[3] Cacchione DA, Pratson LF, Ogston AS (2002). The Shaping of Continental Slopes by Internal Tides. Science.

[4] Puig P. Role of internal waves in the generation of nepheloid layers on the northwestern Alboran slope: Implications for continental margin shaping. *Journal of Geophysical Research* **109** (2004).

[5] Ma X, Yan J, Hou Y, Lin F, Zheng X (2016). Footprints of obliquely incident internal solitary waves and internal tides near the shelf break in the northern South China Sea: Footprints of ISWS and internal tides. Journal of Geophysical Research: Oceans.

[6] Reeder DB, Ma BB, Yang YJ (2011). Very large subaqueous sand dunes on the upper continental slope in the South China Sea generated by episodic, shoaling deep-water internal solitary waves. Marine Geology.

[7] Southard, J. B. & Cacchione, D. A. Experiments on bottom sediment movement by breaking internal waves. Shelf Sediment Transport: Process and Pattern. Dowden, Hutchinson and Ross, Inc., *Stroudsburg, PA, pp*. 83–97 (1972).

[8] Ribbe J, Holloway PE (2001). A model of suspended sediment transport by internal tides. Continental Shelf Research.

[9] Lien R-C, Henyey F, Ma B, Yang YJ (2014). Large-Amplitude Internal Solitary Waves Observed in the Northern South China Sea: Properties and Energetics. Journal of Physical Oceanography.

[10] Tian Z (2017). Experimental investigation of slope sediment resuspension characteristics and influencing factors beneath the internal solitary wave-breaking process. Bulletin of Engineering Geology & the Environment.

[11] Hosegood P, van Haren H (2004). Near-bed solibores over the continental slope in the Faeroe-Shetland Channel. Deep-SeaRes. II..

[12] Masunaga E (2015). Mixing and sediment resuspension associated with internal bores in a shallow bay. Continental Shelf Research.

[13] Richards CG, Bourgault D, Galbraith PS, Hay A, Kelley DE (2013). Measurements of shoaling internal waves and turbulence in an estuary. J.Geophys.Res..

[14] Johnson DR, Weidemann A, Pegau WS (2001). Internal tidal bores and bottom nepheloid layers[J]. Continental Shelf Research.

[15] McPhee-Shaw, E. E. Boundary layer intrusions from a sloping bottom: A mechanism for generating intermediate nepheloid layers. *Journal of Geophysical Research* **107** (2002).

[16] Droghei, R. *et al*. The role of Internal Solitary Waves on deep-water sedimentary processes: the case of up-slope migrating sediment waves off the Messina Strait. *Scientific Reports* **6** (2016). 10.1038/srep36376 PMC5093411 27808239

[17] Bogucki, D. J. Internal solitary waves in the Coastal Mixing and Optics 1996 experiment: Multimodal structure and resuspension. *Journal of Geophysical Research* **110** (2005).

[18] Quaresma LS, Vitorino J, Oliveira A, da Silva J (2007). Evidence of sediment resuspension by nonlinear internal waves on the western Portuguese mid-shelf. Marine Geology.

[19] Chen C-Y, Hsu JR-C, Chen H-H, Kuo C-F, Cheng M-H (2007). Laboratory observations on internal solitary wave evolution on steep and inverse uniform slopes. Ocean Engineering.

[20] Klymak JM, Moum JN (2003). Internal solitary waves of elevation advancing on a shoaling shelf: internal solitary waves of elevation. Geophysical Research Letters.

[21] Butman B, Alexander PS, Scotti A, Beardsley RC, Anderson SP (2006). Large internal waves in Massachusetts Bay transport sediments offshore. Continental Shelf Research.

[22] Bogucki D, Dickey T, Redekopp LG (1997). Sediment resuspension and mixing by resonantly generated internal solitary waves[J]. Journal of Physical Oceanography.

[23] Proni JR, Apel JR (1975). On the use of high-frequency acoustics for the study of internal waves and microstructure [J]. Journal of Geophysical Research.

[24] Ramp SR (2004). Internal Solitons in the Northeastern South China Sea Part I: Sources and Deep Water Propagation. Ieee J Oceanic Eng..

[25] Alford MH (2015). The formation and fate of internal waves in the South China Sea. Nature.

[26] Bai Y (2015). Nonlinear internal solitary waves in the northeast South China Sea near Dongsha Atoll using seismic oceanography (in Chinese). Chin Sci Bull.

[27] Chen JX (2016). Geophysical analysis of abnormal seismic (oceanography) reflection characteristics of oceanic bottom boundary layer. Chinese Journal of Geophysics.

[28] Durrieu de Madron X (2017). Deep sediment resuspension and thick nepheloid layer generation by open-ocean convection. Journal of Geophysical Research: Oceans,.

[29] McCave IN (1986). Local and global aspects of the bottom nepheloid layers in the world ocean. Netherlands Journal of Sea Research.

[30] Bourgault D (2014). Sediment resuspension and nepheloid layers induced by long internal solitary waves shoaling orthogonally on uniform slopes. Cont Shelf Res..

[31] Zhao Y (2015). *In situ* observation of contour currents in the northern South China Sea: Applications for deepwater sediment transport. Earth and Planetary Science Letters.

[32] Xu J, Xie J, Chen Z, Cai S, Long X (2012). Enhanced mixing induced by internal solitary waves in the South China Sea. Continental Shelf Research.

[33] Olsthoorn, J., Stastna, M., & Soontiens, N. Fluid circulation and seepage in lake sediment due to propagating and trapped internal waves: SEEPAGE IN LA`KE SEDIMENT. *Water Resources Research* **48**(11) (2012).

[34] Garrett C, Kunze E (2007). Internal Tide Generation in the Deep Ocean. Annual Review of Fluid Mechanics.

[35] Azetsu-Scott K, Johnson BD, Petrie B (1995). An intermittent, intermediate nepheloid layer in Emerald Basin, Scotian Shelf. Continental Shelf Research.

[36] Zhang X (2018). Polarity variations of internal solitary waves over the continental shelf of the northern South China Sea: impacts of seasonal stratification, mesoscale eddies and internal tides. Journal of Physical Oceanography.

[37] Huang, X. Study on the Spatial Distributions and Temporal Variations of Internal Solitary Waves in the South China Sea. Doctoral dissertation. *Ocean University of China* (2013).

[38] Huang X, Zhao W, Tian J, Yang Q (2014). Mooring observations of internal solitary waves in the deep basin west of Luzon Strait. Acta Oceanologica Sinica.

[39] Syvitski JPM, Kettner A (2011). Sediment flux and the Anthropocene. Philosophical Transactions of the Royal Society A: Mathematical. Physical and Engineering Sciences.

[40] Overeem I (2017). Substantial export of suspended sediment to the global oceans from glacial erosion in Greenland. Nature Geoscience.

[41] Liu JP (2009). Fate of Sediments Delivered to the Sea by Asian Large Rivers: Long-Distance Transport and Formation of Remote Alongshore Clinothems. The Sedimentary Record.

[42] Liu JP (2008). Flux and fate of small mountainous rivers derived sediments into the Taiwan Strait. Marine Geology.

[43] Diercks A-R (2018). Scales of seafloor sediment resuspension in the northern Gulf of Mexico. Elem Sci Anth.

[44] Li X, Zhao Z, Pichel WG (2008). Internal solitary waves in the northwestern South China Sea inferred from satellite images[J]. Geophysical Research Letters.

[45] Sawyer DE, Mason RA, Cook AE, Portnov A (2019). Submarine Landslides Induce Massive Waves in Subsea Brine Pools. Scientific Reports.

