# Author Correction: Deep-sea Sediment Resuspension by Internal Solitary Waves in the Northern South China Sea

**DOI:** 10.1038/s41598-019-53956-y

**Published:** 2019-11-21

**Authors:** Yonggang Jia, Zhuangcai Tian, Xuefa Shi, J. Paul Liu, Jiangxin Chen, Xiaolei Liu, Ruijie Ye, Ziyin Ren, Jiwei Tian

**Affiliations:** 1 Shandong Provincial Key Laboratory of Marine Environment and Geological Engineering, Ocean University of China, Qingdao, 266100 P.R. China; 2 Laboratory for Marine Geology, Qingdao National Laboratory for Marine Science and Technology, Qingdao, 266061 P.R. China; 3 Key Laboratory of Marine Sedimentology and Environmental Geology, First Institute of Oceanography, State Oceanic Administration, Qingdao, Shandong 266061 China; 4 Department of Marine, Earth and Atmospheric Sciences, North Carolina State University, Raleigh, NC 27695 USA; 5 The Key Laboratory of Gas Hydrate, Ministry of Natural Resources, Qingdao Institute of Marine Geology, Qingdao, 266071 P.R. China; 6 Physical Oceanography Laboratory/CIMST, Ocean University of China, Qingdao, 266100 P.R. China; 7 Key Laboratory of Marine Environment & Ecology, Ministry of Education, Qingdao, 266100 P.R. China

Correction to: *Scientific Reports* 10.1038/s41598-019-47886-y, published online 20 August 2019

The original version of this Article contained a typographical error in the Abstract.

“Specifically, we estimated that ISWs could induce and suspend 78.7 Mt/yr of sediment from shelf to deep-sea areas of the northern South China Sea.”

now reads:

“Specifically, we estimated that ISWs could induce and suspend 787 Mt/yr of sediment from shelf to deep-sea areas of the northern South China Sea.”

In addition, the original version of this Article contained a typographical error in the Conclusion.

“We estimated that ISWs could suspend 78.7 Mt/yr sediment from the shelf to the deep sea in the northern South China Sea, which accounted for 6.1% (16.4% of the contribution of Asian rivers) of the global river-discharged sediment transported to the sea.”

now reads:

“We estimated that ISWs could suspend 787 Mt/yr sediment from the shelf to the deep sea in the northern South China Sea, which accounted for 6.1% (16.4% of the contribution of Asian rivers) of the global river-discharged sediment transported to the sea.”

This has now been corrected in the PDF and HTML versions of the Article.

