# Supplementary material for: Deep-sea Sediment Resuspension by Internal Solitary Waves in the Northern South China Sea
Source: Sci Rep. 2019 Aug 20;9:12137. doi: 10.1038/s41598-019-47886-y (PMC6702154; doi:10.1038/s41598-019-47886-y)
Supplement: Supplementary file 1 — supplementary [file 41598_2019_47886_MOESM1_ESM.docx]

**Supplementary**

**Deep-sea Sediment Resuspension by Internal Solitary Waves in the Northern South China Sea**

**Yonggang Jia^1,2,7^, Zhuangcai Tian^1,2^, Xuefa Shi^2,3^, J. Paul Liu^4^, Jiangxin Chen^2,5^, Xiaolei Liu^1,2,7^, Ruijie Ye^2,6^, Ziyin Ren^1,2^, Jiwei Tian^2,6*^**

^1^Shandong Provincial Key Laboratory of Marine Environment and Geological Engineering, Ocean University of China, Qingdao 266100, P. R. China

^2^Laboratory for Marine Geology, Qingdao National Laboratory for Marine Science and Technology, Qingdao 266061, P. R. China

^3^Key Laboratory of Marine Sedimentology and Environmental Geology, First Institute of Oceanography,

State Oceanic Administration, Qingdao, Shandong 266061, China

^4^Department of Marine, Earth and Atmospheric Sciences, North Carolina State University, Raleigh, NC 27695, USA

^5^The Key Laboratory of Gas Hydrate, Ministry of Natural Resources, Qingdao Institute of Marine Geology, Qingdao, 266071, P. R. China

^6^Physical Oceanography Laboratory/CIMST, Ocean University of China, Qingdao 266100, P. R. China

^7^Key Laboratory of Marine Environment & Ecology, Ministry of Education, Qingdao 266100, P. R. China

**Abstract.** This document contains supplementary materials including estimates of resuspended sediment by internal solitary waves in the northern South China Sea.

The ubiquitous internal solitary waves (ISWs) are believed to generate in the Luzon Strait and propagate in the WNW direction in the northern South China Sea, eventually dissipating in the shallow coastal sea, spreading more than 600 kilometres (Alford et al., 2015). The ISWs do not occur over the entire shelf of the northern South China Sea (Li et al., 2008). Hence, the hotspot regions of sediment resuspension induced by ISWs include all continental slopes and shelf breaks of the northern South China Sea (Li et al., 2008; Zhao et al., 2014). We used 0.5×0.5° mapping grid to estimate the footprints of ISWs that propagated from the continental slope to the shelf break. The total size of regions of ISWs in the northern South China Sea was approximately 111.12×111.12×cos20.5°×56/4 =1.62×10^5^ km^2^ =1.62×10^11^ m^2^.

The average thickness and maximum concentration of bottom nepheloid layers by ISWs were approximately 100 m and 0.135 mg/l, respectively. The statistical data showed that there were more than 360 ISWs (approximately one ISW per day) every year in the northern South China Sea (Huang 2013; Huang et al., 2014). The estimated total amount of resuspended sediment every year in the northern South China Sea was approximately 1.62×10^11^×100×0.135×10^-3^×360 =7.87×10^11^ kg =7.87×10^8^ tons.


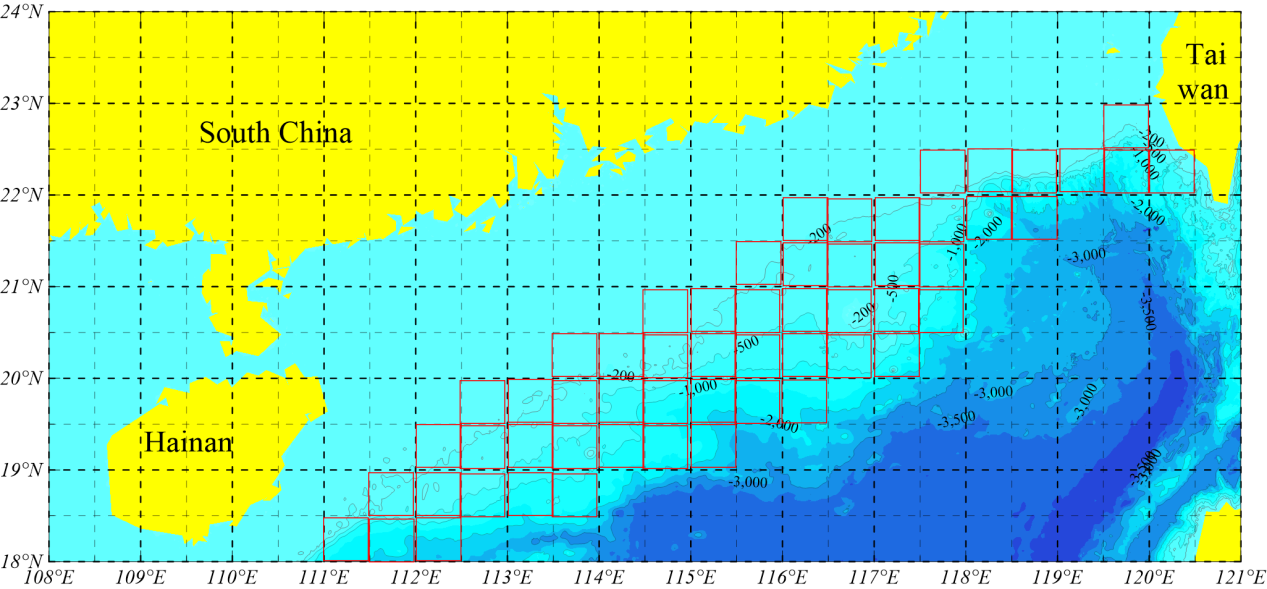


**Fig. S1** Bathymetric map of the northern South China Sea. The red rectangles indicate areas of suspended sediments by ISWs.

**References**

Alford, M. H. et al. (2015). The formation and fate of internal waves in the South China Sea. Nature 521, 65–69.

Huang, X. (2013). Study on the Spatial Distributions and Temporal Variations of Internal Solitary Waves in the South China Sea. Doctoral dissertation. Ocean University of China.

Huang, X., Zhao, W., Tian, J., & Yang, Q. (2014). Mooring observations of internal solitary waves in the deep basin west of Luzon Strait. Acta Oceanologica Sinica, 33(3), 82–89.

Li X, Zhao Z, Pichel W G. (2008), Internal solitary waves in the northwestern South China Sea inferred from satellite images[J]. Geophysical Research Letters, 35(13):344-349.

Zhao, Z., Liu, B., & Li, X. (2014). Internal solitary waves in the China seas observed using satellite remote-sensing techniques: a review and perspectives. International Journal of Remote Sensing, 35(11–12), 3926–3946.
